# Characterization of Thermal Damage Due to Two-Temperature High-Order Thermal Lagging in a Three-Dimensional Biological Tissue Subjected to a Rectangular Laser Pulse

**DOI:** 10.3390/polym12040922

**Published:** 2020-04-16

**Authors:** Hamdy M. Youssef, Najat. A. Alghamdi

**Affiliations:** 1 Engineering Mechanics Department, College of Engineering and Islamic Architecture, Umm Al-Qura University, Makkah-KSA 21955, Saudi Arabia; 2 Mathematics Department, Faculty of Science, Umm Al-Qura University, Makkah-KSA 21955, Saudi Arabia; najatalghamdi@gmail.com

**Keywords:** two-temperature thermal lagging, biological tissue, laser pulse, thermal damage, three dimensions

## Abstract

The use of lasers and thermal transfers on the skin is fundamental in medical and clinical treatments. In this paper, we constructed and applied bioheat transfer equations in the context of a two-temperature heat conduction model in order to discuss the three-dimensional variation in the temperature of laser-irradiated biological tissue. The amount of thermal damage in the tissue was calculated using the Arrhenius integral. Mathematical difficulties were encountered in applying the equations. As a result, the Laplace and Fourier transform technique was employed, and solutions for the conductive temperature and dynamical temperature were obtained in the Fourier transform domain.

## 1. Introduction

Laser pulses have been increasingly used in medical treatments over the past half-century. Thermal damage is essential when considering the quality of therapy. The effects of the laser pulse on biological tissues are numerous, thermal effects being the main ones. Thermal transfer is an essential phenomenon that results in temperature increases due to thermal effects and is a common factor in the production of thermal damage. Due to a lack of data and knowledge regarding thermal responses, clinical doctors cannot adequately control laser power and application time to mitigate this damage, which might cause skin tissue thermal damage in some cases or insufficient thermal dose in other cases. Thermal damage in biological tissues is known to be due to chemical reactions [1,2,3,4].

To predict the amount of thermal damage, it is necessary to examine the thermal process induced by the laser energy source. Consequently, a study of thermal transport is essential for developing an appropriate approach for successful therapy, ensuring maximum patient protection. Experimentation allows recognizing the problems encountered in medical treatments. Obtaining definite conclusions from experimentation can be complicated by the complexity of the type of tissue to be treated, the laser technologies, and the physical and biochemical processes involved. Hence, theoretical approaches using mathematical models have been used to study the effect of heat and the damage caused by laser radiation on the skin tissue [4,5]. Various researchers have studied bioheat transfer in biological tissues. The most well-known examples are studies by Pennes [6], Abramson [7], Lemons, et al. [8], Weinbaum et al. [9], and Xu et al. [10]. The Pennes bioheat transfer equation is usually used to study thermal transportation in human tissues because of its simplicity and validity. It is based on an improvement of Fourier’s law, which describes an infinitely fast propagation of a thermal wave. Regarding thermal behavior in nonhomogeneous media, a period of relaxation is needed in order to combine enough energy to be transferred to the nearest element [11,12]. An accurate description of the thermal behavior in biological tissues is essential to study heat transfer in living tissues. Mitra et al. [13] conducted an experimental study in processed meat and described the thermal relaxation time. In the literature [14,15,16], non-Fourier models of bioheat transfer were reported for studying thermal transportation behaviors in living tissues and to solve the paradox of the instant responses of thermal disturbance which occurred in the Pennes bioheat transfer equation.

Several attempts have been made by researchers to model the complex thermal behavior of the human body [17]. Tzou proposed the dual-phase-lag model (DPL) and introduced a phase lag for the temperature gradient in order to solve the paradox in Fourier conduction and to consider heat transport, which is absent in the thermal wave model [18]. Xu et al. studied the impact of DPL on the biothermal and mechanical behavior of skin [19,20]. The DPL model of bioheat transfer improved the first-order model by using the Taylor series expansion and was used to describe the linear effect of the phase lag times on biological systems and the thermal behavior in biological systems. This linear DPL equation has been used in various theoretical studies [21,22,23]. 

Youssef improved the theory of heat conduction in deformable bodies, which had been previously studied by Chen and Gurtin, and found it to depend upon two distinct temperatures, i.e., the conductive and the thermodynamic temperatures [24,25]. The difference between the conductive and the thermodynamic temperatures is proportional to the heat source, and these two temperatures are identical in the absence of a heat source. Youssef applied the theory of two-temperature heat conduction, providing many examples [26,27,28].

In the present work, a new mathematical model of bioheat transfer was constructed in the context of a two-temperature heat conduction model. The governing equations of the model were applied to three-dimensional biological tissues, whereby the surface of the tissue was subjected to a rectangular laser pulse. The amount of thermal damage was calculated using the Arrhenius integral. 

## 2. Problem Formulation

**Figure 1:**
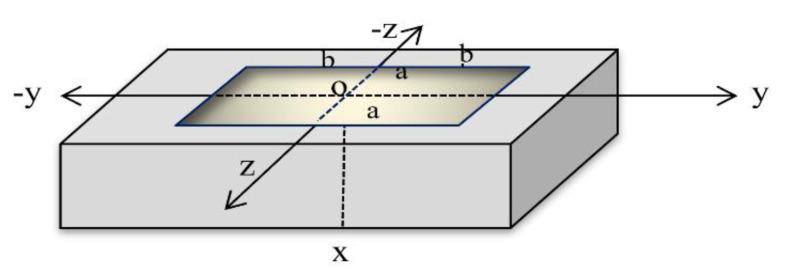
The three-dimensional skin tissue.

Consider a homogeneous skin tissue sample in three dimensions, which is defined as Ψ={x,y,z:  0≤x<∞, −∞<y<∞, −∞<z<∞}. The skin is considered to be initially resting and is shocked by a rectangular pulse of a laser at the bounding plane on the surface x=0 [28,29,30], as in Figure 1.

Tzou proposed the DPL model and the effect of microstructural interactions [18], described by

(1) $$q\left(x,y,z,t+\tau_{q}\right)=-K\nabla T\left(x,y,z,t+\tau_{T}\right)$$

where $\tau_{T},\tau_{q}$ are the phase lags of the temperature gradient and the phase lag of the heat flux, respectively; in general, the relaxation times $\tau_{T},\tau_{q}$ have small values, but in biological materials, these parameters have significant impacts. *T* is the temperature, *q* is the heat flux, *K* is the thermal conductivity, and *t* is time. 

The energy conservation of the bioheat transfer equation is described in [1,5,18,21]:

(2) $$\rho C\frac{\partial T}{\partial t}=-\nabla \cdot q-W_{b}C_{b}\rho_{p}\left(T-T_{b}\right)+\left(Q_{met}+Q_{ext}\right)$$

where C is specific heat, ρ is density, and $C_{b}$ and $W_{b}$ are the specific heat and perfusion rates of blood, respectively. $Q_{met}$ is metabolic heat generation and is constant, while $Q_{ext}$ is the heat source for spatial heating and may vary; $T_{b}$ is arterial temperature, which is constant. The term $W_{b}C_{b}\left(T_{D}-T_{b}\right)$ expresses the heating due to convection within the head per unit mass of the tissue and is assumed to be homogenous.

Youssef considered the two-temperature model of heat conduction to differentiate between the conductive temperature and the dynamical temperature [25,28]:

(3) $$q\left(x,y,z,t+\tau_{q}\right)=-K\nabla T_{C}\left(x,y,z,t+\tau_{T}\right)$$

(4) $$T_{C}-T_{D}=\beta \;\nabla^{2}T_{C}$$

where β≥0 is the two-temperature parameter and is constant, $T_{C}$ is the conductive temperature, while $T_{D}$ is the dynamical temperature. 

The energy conservation equation of bioheat transfer in the context of the two-temperature model takes the form

(5) $$\rho C\frac{\partial T_{D}}{\partial t}=-\nabla \cdot q-W_{b}C_{b}\rho_{p}\left(T_{D}-T_{b}\right)+\left(Q_{met}+Q_{ext}\right)$$

By applying the second-order of Taylor expansion, Equation (3) for heat conduction takes the form

(6) $$\left(1+\tau_{q}\frac{\partial }{\partial t}+\frac{\tau_{q}^{2}}{2}\frac{\partial^{2}}{\partial \;t^{2}}\right)q=-K\left(1+\tau_{T}\frac{\partial }{\partial t}+\frac{\tau_{T}^{2}}{2}\frac{\partial^{2}}{\partial \;t^{2}}\right)\nabla T_{C}$$

Thus, we have

(7) $$\left(1+\tau_{q}\frac{\partial }{\partial t}+\frac{\tau_{q}^{2}}{2}\frac{\partial^{2}}{\partial \;t^{2}}\right)\nabla \cdot q=-K\left(1+\tau_{T}\frac{\partial }{\partial t}+\frac{\tau_{T}^{2}}{2}\frac{\partial^{2}}{\partial \;t^{2}}\right)\nabla^{2}T_{C}$$

Equation (5) can be rewritten in the following form:

(8) $$\begin{array}{l} \rho C\left(1+\tau_{q}\frac{\partial }{\partial t}+\frac{\tau_{q}^{2}}{2}\frac{\partial^{2}}{\partial \;t^{2}}\right)\frac{\partial T_{D}}{\partial t}=-\left(1+\tau_{q}\frac{\partial }{\partial t}+\frac{\tau_{q}^{2}}{2}\frac{\partial^{2}}{\partial \;t^{2}}\right)\nabla \cdot q-\\ \;W_{b}C_{b}\rho_{p}\left(1+\tau_{q}\frac{\partial }{\partial t}+\frac{\tau_{q}^{2}}{2}\frac{\partial^{2}}{\partial \;t^{2}}\right)\left(T_{D}-T_{b}\right)+\left(1+\tau_{q}\frac{\partial }{\partial t}+\frac{\tau_{q}^{2}}{2}\frac{\partial^{2}}{\partial \;t^{2}}\right)\left(Q_{met}+Q_{ext}\right) \end{array}$$

By eliminating the term $\;\left(1+\tau_{q}\frac{\partial }{\partial t}+\frac{\tau_{q}^{2}}{2}\frac{\partial^{2}}{\partial \;t^{2}}\right)\nabla \cdot q$ from Equations (7) and (8), we get

(9) $$\begin{array}{l} K\left(1+\tau_{T}\frac{\partial }{\partial t}+\frac{\tau_{T}^{2}}{2}\frac{\partial^{2}}{\partial \;t^{2}}\right)\nabla^{2}T_{C}\;=\rho C\left(1+\tau_{q}\frac{\partial }{\partial t}+\frac{\tau_{q}^{2}}{2}\frac{\partial^{2}}{\partial \;t^{2}}\right)\frac{\partial T_{D}}{\partial t}+\\ \;W_{b}C_{b}\rho_{p}\left(1+\tau_{q}\frac{\partial }{\partial t}+\frac{\tau_{q}^{2}}{2}\frac{\partial^{2}}{\partial \;t^{2}}\right)\left(T_{D}-T_{b}\right)-\left(1+\tau_{q}\frac{\partial }{\partial t}+\frac{\tau_{q}^{2}}{2}\frac{\partial^{2}}{\partial \;t^{2}}\right)\left(Q_{met}+Q_{ext}\right) \end{array}$$

Consider that φ is the conductive temperature increment and θ is the dynamical temperature increment, as follows [28]:

(10) $$\varphi \left(x,y,z,t\right)=T_{C}\left(x,y,z,t\right)-T_{b},\;\;\theta \left(x,y,z,t\right)=T_{D}\left(x,y,z,t\right)-T_{b}$$

From Equations (9) and (10), we get

(11) $$\begin{array}{l} K\left(1+\tau_{T}\frac{\partial }{\partial t}+\frac{\tau_{T}^{2}}{2}\frac{\partial^{2}}{\partial \;t^{2}}\right)\left(\frac{\partial^{2}\varphi }{\partial x^{2}}+\frac{\partial^{2}\varphi }{\partial y^{2}}+\frac{\partial^{2}\varphi }{\partial z^{2}}\right)=\rho C\left(1+\tau_{q}\frac{\partial }{\partial t}+\frac{\tau_{q}^{2}}{2}\frac{\partial^{2}}{\partial \;t^{2}}\right)\frac{\partial \theta }{\partial t}+\\ \;W_{b}C_{b}\rho_{p}\left(1+\tau_{q}\frac{\partial }{\partial t}+\frac{\tau_{q}^{2}}{2}\frac{\partial^{2}}{\partial \;t^{2}}\right)\theta -\left(1+\tau_{q}\frac{\partial }{\partial t}+\frac{\tau_{q}^{2}}{2}\frac{\partial^{2}}{\partial \;t^{2}}\right)\left(Q_{met}+Q_{ext}\right) \end{array}$$

and

(12) $$\theta =\varphi -\beta \left(\frac{\partial^{2}\varphi }{\partial x^{2}}+\frac{\partial^{2}\varphi }{\partial y^{2}}+\frac{\partial^{2}\varphi }{\partial z^{2}}\right)$$

The metabolic heat source is efficient due to the chemical reactions, and it is constant: $Q_{met}$ = 368.1 W/m^3^.

When applying the thermal shock from the laser beam on the skin surface, the laser energy will be scattered, absorbed, and transmitted. Lambert expressed the laser power intensity in relation to tissue depth using Beer’s law, as follows [2]:

(13) $$I\left(x,y,z,t\right)=I_{0}e^{-\delta x}H\left(t\right)H\left(a-\left|y\right|\right)H\left(b-\left|z\right|\right)$$

We neglect the scattering in heat generation; therefore, the specific absorption rate in the skin tissue can be expressed as follows [28]:

(14) $$Q_{ext}\left(x,y,z,t\right)=-\frac{\partial I}{\partial x}=\delta I_{0}e^{-\delta x}H\left(t\right)H\left(a-\left|y\right|\right)H\left(b-\left|z\right|\right)$$

In Equation (13), $I_{0}$ (W/m^2^) represents the power density of the laser pulse, and H(t) is the unit step Heaviside function. The parameter δ scales the penetration depth, which gives the value of how deep the laser heat wave can penetrate through the skin tissue. 

The rectangular thermal pulse of the laser pulse acts on a bandwidth of 2a centered on the *y*-axis and bandwidth of 2b centered around the *z*-axis on the surface of the half-space x=0, and is zero elsewhere, as in Figure 1. Hence, we get

(15) $$\begin{array}{l} K\left(1+\tau_{T}\frac{\partial }{\partial t}+\frac{\tau_{T}^{2}}{2}\frac{\partial^{2}}{\partial \;t^{2}}\right)\left(\frac{\partial^{2}\varphi }{\partial x^{2}}+\frac{\partial^{2}\varphi }{\partial y^{2}}+\frac{\partial^{2}\varphi }{\partial z^{2}}\right)\;\;=\rho C\left(1+\tau_{q}\frac{\partial }{\partial t}+\frac{\tau_{q}^{2}}{2}\frac{\partial^{2}}{\partial \;t^{2}}\right)\frac{\partial \theta }{\partial t}+\\ \;W_{b}C_{b}\rho_{p}\left(1+\tau_{q}\frac{\partial }{\partial t}+\frac{\tau_{q}^{2}}{2}\frac{\partial^{2}}{\partial \;t^{2}}\right)\theta \\ \;-(1+\tau_{q}\frac{\partial }{\partial t}+\frac{\tau_{q}^{2}}{2}\frac{\partial^{2}}{\partial \;t^{2}})\left(1I_{0}e^{-\delta x}H\left(t\right)H\left(a-\left|y\right|\right)H\left(b-\left|z\right|\right)+Q_{met}\right) \end{array}$$

By applying the Laplace transform for Equations (12) and (15), we get

(16) $$\bar{f}\left(x,y,z;s\right)=\int_{0}^{\infty }f\left(x,y,z;t\right)e^{-st}dt$$

where the initial conditions are

(17) $${\varphi \left(x,t\right)|}_{t=0}={\theta \left(x,t\right)|}_{t=0}={\frac{\partial \varphi \left(x,t\right)}{\partial t}|}_{t=0}={\frac{\partial \theta \left(x,t\right)}{\partial t}|}_{t=0}=0$$

Thus, we get

(18) $$\begin{array}{c} K\;h_{T}\left(\frac{\partial^{2}\bar{\varphi }}{\partial x^{2}}+\frac{\partial^{2}\bar{\varphi }}{\partial y^{2}}+\frac{\partial^{2}\bar{\varphi }}{\partial z^{2}}\right)\;\;=h_{q}\left(\rho C\;s+W_{b}C_{b}\rho_{p}\right)\bar{\theta }-\\ \;\frac{h_{q}\delta I_{0}}{s}H\left(a-\left|y\right|\right)H\left(b-\left|z\right|\right)e^{-\delta x}-\frac{Q_{met}\;h_{q}}{s} \end{array}$$

and

(19) $$\bar{\theta }=\bar{\varphi }-\beta \left(\frac{\partial^{2}\bar{\varphi }}{\partial x^{2}}+\frac{\partial^{2}\bar{\varphi }}{\partial y^{2}}+\frac{\partial^{2}\bar{\varphi }}{\partial z^{2}}\right)$$

where $h_{T}=\left(1+s\tau_{T}-\frac{s^{2}\tau_{T}^{2}}{2}\right),\;\;\;h_{q}=\left(1+s\tau_{q}-\frac{s^{2}\tau_{q}^{2}}{2}\right)$.

## 3. The Governing Equations in the Fourier Transform Domain

Use the double Fourier transform for any function f(x,y,z;s), defined as follows:

(20) $$F\left[\bar{f}\left(x,y,z;s\right)\right]=\tilde{\bar{f}}(x,p,q;s)=\frac{1}{2\pi }\int_{-\infty }^{\infty }\int_{-\infty }^{\infty }\bar{f}(x,y,z;s)\;e^{-i\left(py+qz\right)}\;dy\;dz$$

where the inversion transform of the double Fourier transform takes the form

(21) $$F^{-1}\left[\tilde{\bar{f}}\left(x,p,q;s\right)\right]=\bar{f}(x,y,z;s)=\frac{1}{2\pi }\int_{-\infty }^{\infty }\int_{-\infty }^{\infty }\tilde{\bar{f}}(x,p,q;s)\;e^{i\left(py+qz\right)}dp\;dq$$

Thus, we have

(22) $$F\left[\nabla^{2}\bar{f}\left(x,y,z;s\right)\right]=\left(\frac{d^{2}}{d\;x^{2}}-p^{2}-q^{2}\right)\tilde{\bar{f}}\left(x,p,q;s\right)$$

Hence, we get

(23) $$\begin{array}{c} \left(\frac{\partial^{2}\tilde{\bar{\varphi }}}{\partial x^{2}}-p^{2}\tilde{\bar{\varphi }}-q^{2}\tilde{\bar{\varphi }}\right)\;\;=\frac{h_{q}\left(\rho C\;s+W_{b}C_{b}\rho_{p}\right)}{K\;h_{T}}\tilde{\bar{\theta }}-\\ \;\frac{2\delta I_{0}h_{q}\sin\left(pa\right)\sin\left(qb\right)}{\pi \;s\;pqK\;h_{T}}e^{-\delta x}\;-\frac{Q_{met}\;h_{q}\delta \left(p\right)\delta \left(q\right)}{sK\;h_{T}} \end{array}$$

and

(24) $$\tilde{\bar{\theta }}=\left[1+\beta \left(p^{2}+q^{2}\right)\right]\tilde{\bar{\varphi }}-\beta \frac{\partial^{2}\tilde{\bar{\varphi }}}{\partial x^{2}}$$

By eliminating $\tilde{\bar{\theta }}$ from Equations (23) and (24), we obtain the following differential equation:

(25) $$\frac{\partial^{2}\tilde{\bar{\varphi }}\left(x,s\right)}{\partial x^{2}}\;-\lambda^{2}\tilde{\bar{\varphi }}\left(x,s\right)=-k_{1}e^{-1x}-k_{2},\;\;\;\;0\leq x\leq L$$

where $\lambda^{2}=\frac{h_{q}\left(\rho C\;s+W_{b}C_{b}\rho_{p}\right)\left(1+\beta \left(p^{2}+q^{2}\right)\right)+Kh_{T}\left(p^{2}+q^{2}\right)}{\left[K\;h_{T}\;+\beta h_{q}\left(\rho C\;s+W_{b}C_{b}\rho_{p}\right)\right]}$,

$k_{1}=\frac{2\delta I_{0}\sin\left(pa\right)\sin\left(qb\right)h_{q}\;\;}{\pi \;s\;pq\left(K\;h_{T}\;+\beta h_{q}\left(\rho C\;s+W_{b}C_{b}\rho_{p}\right)\right)}$ and $k_{2}=\frac{Q_{met}\;\delta \left(p\right)\delta \left(q\right)h_{q}}{s\left(K\;h_{T}\;+\beta h_{q}\left(\rho C\;s+W_{b}C_{b}\rho_{p}\right)\right)}$.

The solution to the differential Equation (25), in general form, is

(26) $$\tilde{\bar{\varphi }}\left(x,p,q,s\right)=c_{1}e^{\lambda x}+c_{2}e^{-\lambda x}-\frac{k_{1}}{\delta^{2}-\lambda^{2}}e^{-\delta x}+\frac{k_{2}}{\lambda^{2}},\;\;\;\;\;0\leq x\leq L$$

where $c_{1}$ and $c_{2}$ are some parameters that have to be determined.

Apply the following boundary conditions:

(27) $${\frac{\partial \tilde{\bar{\varphi }}\left(x,p,q,s\right)}{\partial x}|}_{x=0}=-Q_{ext}\left(0,y,z,t\right)=-q_{0},\;\;\;{\frac{\partial \tilde{\bar{\varphi }}\left(x,p,q,s\right)}{\partial x}|}_{x=L}=0$$

Hence, we obtain

(28) $$\begin{array}{c} \tilde{\bar{\varphi }}\left(x,p,q,s\right)=k_{3}\cosh\lambda \left(L-x\right)-k_{4}\cosh\lambda x-\;\;\\ \;\frac{k_{1}}{\left(\delta^{2}-\lambda^{2}\right)}e^{-\delta x}+\frac{k_{2}}{\lambda^{2}},\;\;\;\;\;0\leq x\leq L \end{array}$$

where $k_{3}=\frac{1}{\lambda \sinh\lambda L}\left(\frac{\delta k_{1}}{\left(\delta^{2}-\lambda^{2}\right)}+q_{0}\right),\;\;\;k_{4}=\frac{\delta k_{1}e^{-\delta L}}{\lambda \left(\delta^{2}-\lambda^{2}\right)\sinh\lambda L}$.

## 4. Thermal Damage

After the well-known works of Henriques and Moritz, many researchers have proposed some other models with a similar format where the only differences are in the coefficients used in the burn damage integral due to the different experimental databases used to define the models and the different emphasis given to the damage when analyzing the burn process. 

In this work, we considered $E_{a}/R$ = 55000 K^−1^, and the frequency factor took the value A = 3.1 × 10^98^ s^−1^ [4,23].

Thus, thermal damage for the skin tissue was calculated by the Arrhenius integral formula:

(29) $$\Omega_{\;}=A\int_{0}^{t}e^{\frac{-E_{a}/R}{\;T_{D}\left(x,y,z,\xi \right)}}d\xi$$

where $T_{D}=\left(\theta +37.0+273.0\right)K$.

## 5. Results and Discussions

To solve the problem in the physical domain, we must find the inverse of the double Fourier and Laplace transforms in Equation (28). All the parameters may be formally expressed as functions of x, and the parameters of the Fourier and Laplace transforms *q*, *p*, and *s* may be expressed as functions of the form $\tilde{\bar{f}}\left(x,q,p;s\right)$.

First, we found the inverse of the double Fourier transform using Equation (21). That gave the expression $\bar{f}\left(x,y,z;s\right)$ in the Laplace transform domain.

(30) $$\begin{array}{cc} \bar{f}(x,y,z;s) & =F^{-1}\left[\tilde{\bar{f}}\left(x,p,q;s\right)\right]\\ & =\frac{1}{2\pi }\int_{-\infty }^{\infty }\;\int_{-\infty }^{\infty }\tilde{\bar{f}}(x,p,q;s)\;e^{i\left(py+qz\right)}\;dp\;dq\\ & =\frac{2}{\pi }\;\;\int_{0}^{\infty }\;\int_{0}^{\infty }\left[\cos\;\left(p\;y+qz\right)\;{\tilde{\bar{f}}}_{e}\;+i\;\sin\left(p\;y+qz\right)\;{\tilde{\bar{f}}}_{o}\right]dp\;dq \end{array}$$

where ${\tilde{\bar{f}}}_{e}\;$ and ${\tilde{\bar{f}}}_{o}$ denote the even and the odd parts of the function $\tilde{\bar{f}}\left(x,q,p;s\right)$, respectively [28].

To obtain the inversion of the Laplace transform, the Riemann sum approximation method was used. In this method, any function in the Laplace domain may be inverted to the time domain [18,28]:

(31) g(t)=eκtt[12g¯(κ)+Re∑n=1N(−1)ng¯(κ+ nπIt)]

where *R*_e_ is the real part and $I=\sqrt{-1}$ is the imaginary number unit. 

For faster convergence, various numerical experiments have shown that the parameter κ must satisfy the relationship κ t≈4.7 [18].

The values of the relevant thermal parameters used in the present calculations are shown in Table 1 [4,10,19,23].

**Figure 2:**
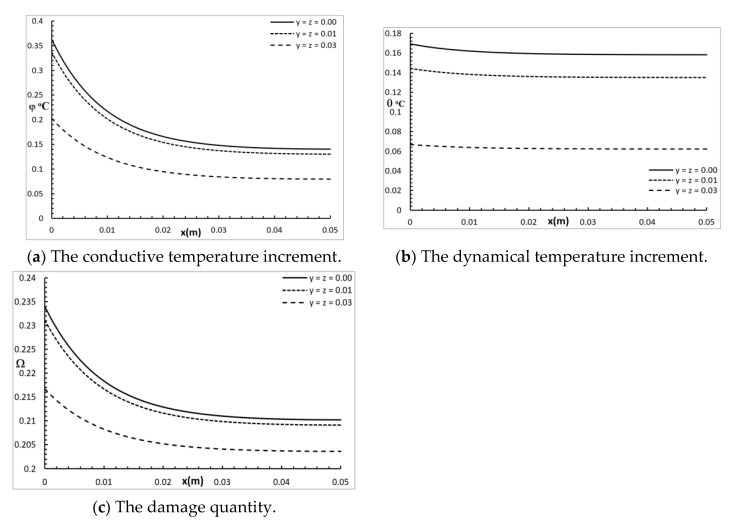
The studied functions at various positions along the axes.

Figure 2 shows the conductive temperature distribution, the dynamical temperature distribution, and the thermal damage distribution for a wide range of values on the *x*-axis (0.0≤x≤0.05)m and at various positions of y and z (0.0,0.01,0.03)m t=30.0 s. The values of both y and z have significant effects on all the studied functions, and increasing the values of y and z causes a decrease in the values of all the studied functions. The values of the conductive temperature increment on the bounding plane of the surface were φ(0,0,0;30.0s) = 0.36 °C φ(0,0.01,0.01;30.0s) = 0.34 °C, and φ(0,0.03,0.03;30.0s) = 0.20 °C. The values of the dynamical temperature increment on the bounding plane of the surface were θ(0,0,0;30.0s) = 0.17 °C θ(0,0.01,0.01;30.0s) = 0.145 °C, and θ(0,0.03,0.03;30.0s) = 0.068 °C. Thus, the values of the dynamical temperature increment were smaller than the values of the conductive temperature increment in the same positions. The values of the damage quantities on the bounding plane of the surface were Ω(0,0,0;30.0s)=0.234 Ω(0,0.01,0.01;30.0s)=0.231, and Ω(0,0.03,0.03;30.0s)=0.217.

**Figure 3:**
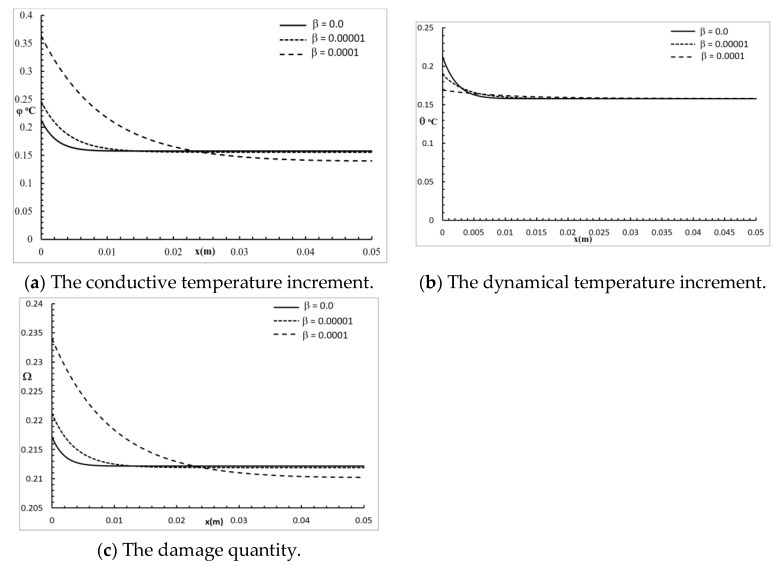
The studied functions at various positions for various values of the two-temperature parameter.

Figure 3 shows the conductive temperature increment, the dynamical temperature increment, and the damage distribution for a wide range of values on the *x*-axis (0.0≤x≤0.05)m and at the position of y=z=0.0, with t=30.0 s, and various values of the two-temperature parameter $\beta =\left(0.0,10^{-5},10^{-4}\right)$. The two-temperature parameter had a significant effect on all the studied functions. Increasing the value of the two-temperature parameter caused an increase in the values of the conductive temperature increment up to the position x=0.0225 m; after that, the three curves were close. Increasing the value of the two-temperature parameter caused an increase in the values of the dynamical temperature increment up to the position x=0.005 m; after that, the three curves were highly similar. Increasing the value of the two-temperature parameter caused an increase in the values of the damage quantity up to the position x=0.0225 m; after that, the three curves were close.

**Figure 4:**
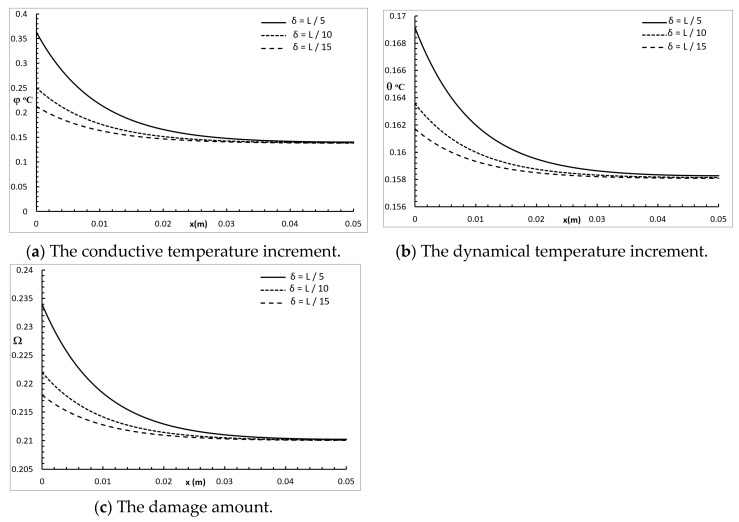
The studied functions for various values of the penetration depth parameter.

Figure 4 represents the conductive temperature increment, the dynamical temperature increment, and the damage distribution for a wide range of values on the *x*-axis (0.0≤x≤0.05)m and various values of the penetration depth parameter δ=(L/5,L/10,L/15) when y=z=0.0 and t=30.0 s. The penetration depth parameter had a significant effect on all the studied functions. Increasing the value of the penetration depth parameter caused an increase in the value of the conductive temperature increment, up to the position x=0.038 m, and after that, the three cases were highly similar. Increasing the value of the penetration depth parameter caused an increase in the value of the dynamical temperature increment up to the end position x=0.05 m. Increasing the value of the penetration depth parameter caused an increase in the value of the damage amount up to the position x=0.038 m, and after that, the three cases were highly similar.

**Figure 5:**
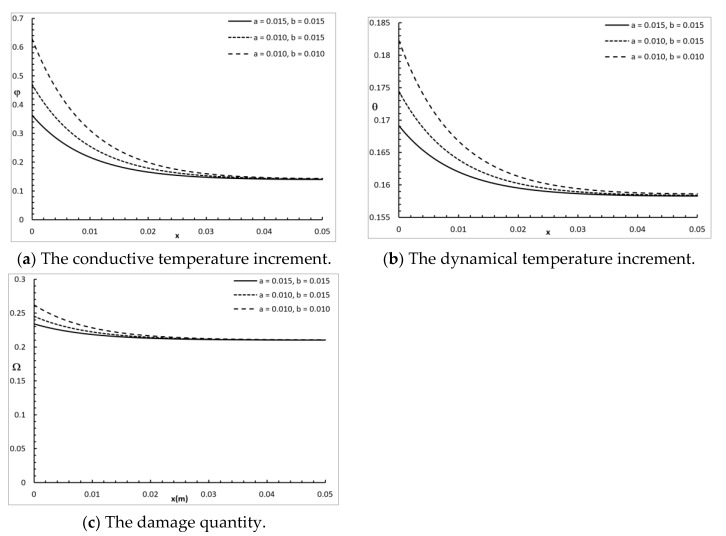
The studied functions for various values of the rectangular laser pulse.

Figure 5 shows the conductive temperature increment, the dynamical temperature increment, and the damage amount distribution for a wide range of values on the *x*-axis (0.0≤x≤0.05)m and various rectangular laser pulses a,b=(0.010,0.015)m, when y=z=0.0 and t=30.0 s. The dimensions of the rectangular laser pulse had a significant effect on the conductive temperature increment, the dynamical temperature increment, and the damage amount distribution. Increasing the values of a and b decreased the value of the conductive temperature increment up to the position x=0.035 m, and after that, the three cases were highly similar. Increasing the values of a and b decreased the value of the dynamical temperature increment up to the position x=0.04 m, and after that, the three cases were highly similar. Increasing the values of a and b decreased the value of the damage amount up to the position x=0.023 m, and after that, the three cases were highly similar.

**Figure 6:**
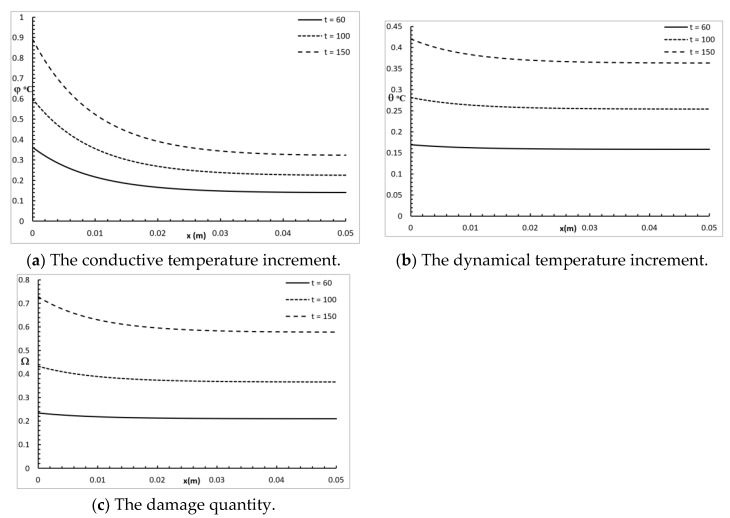
The studied functions for various values of the time t.

Figure 6 represents the conductive temperature increment, the dynamical temperature increment, and the damage amount distribution for a wide range of values on the *x*-axis (0.0≤x≤0.05)m and various values for the time t=(60,100,150)s when y=z=0.0. The time had a significant effect on the conductive temperature increment, the dynamical temperature increment, and the damage amount distribution. Increasing the value of the time caused an increase in the conductive temperature increment, the dynamical temperature increment, and the damage amount distribution up to the end x=0.05 m.

**Figure 7:**
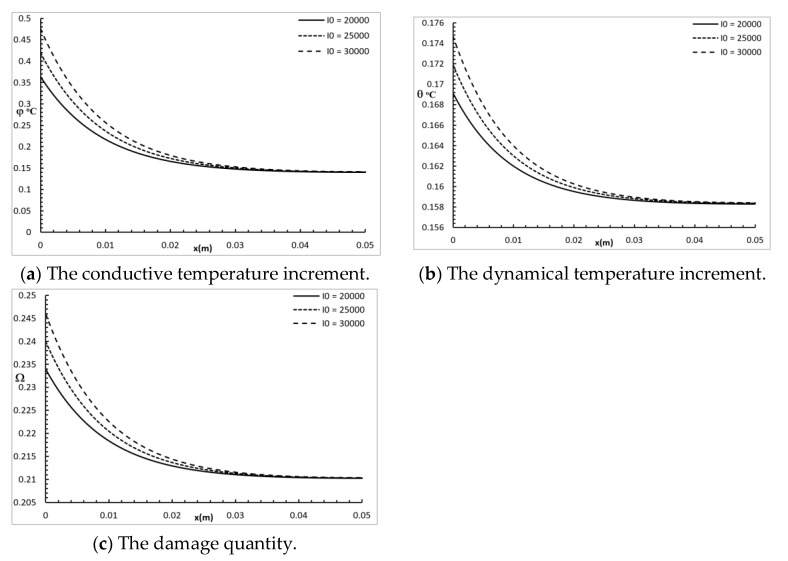
The studied functions for various values of the power density parameter.

Figure 7 shows the conductive temperature increment, the dynamical temperature increment, and the damage quantity distribution for a wide range of values on the *x*-axis (0.0≤x≤0.05)m and various times $I_{0}=\left(2,2.5,3\right)$ × 10^3^ W/m^2^, when y=z=0.0 and t=30.0 s. The power density of the laser pulse had a significant effect on the conductive temperature increment, the dynamical temperature increment, and the damage amount distribution. Increasing the power density of the laser pulse caused an increase in the values of the conductive temperature increment up to the position x=0.025 m, and after that, the three cases were highly similar. Increasing the power density of laser pulse caused an increase in the values of the dynamical temperature increment up to the position x=0.03 m, and after that, the three cases were highly similar. Increasing the power density of the laser pulse caused an increase in the values of damage amount up to the position x=0.025 m, and after that, the three cases were highly similar.

## 6. Conclusions

In this paper, a two-temperature bioheat transfer model was constructed and used to discuss the three-dimensional variation in the temperature of a skin tissue sample subjected to an irradiated rectangular laser pulse. The conductive temperature increment, the dynamical temperature increment, and the damage amount were calculated using the Arrhenius integral.

The results showed that the x, y, and z positions, the two-temperature parameter, the dimensions of the rectangular laser pulse, the penetration depth parameter, the time, and the power density of the laser pulse had significant impacts on the conductive temperature, dynamical temperature, and amount of thermal damage. Increasing the two-temperature parameter, penetration depth parameter, time, and power density of the laser pulse increased the conductive temperature increment, the dynamical temperature increment, and the amount of damage. Increasing the value of the position and the dimensions of the rectangular laser pulse decreased the conductive temperature increment, dynamical temperature increment, and the amount of damage.

## Figures and Tables

**Table 1:** Material properties of the skin tissue employed.

Parameter	Unit	Skin Tissue
K	W/m °C	0.628
ρ	kg/m^3^	1000
$\rho_{b}$	kg/m^3^	1060
C	J/kg °C	4187
$C_{b}$	J/kg °C	3860
$W_{b}$	ml/C m	0.00187
$T_{b}$	°C	37
$\tau_{T}$	*s*	10
$\tau_{q}$	*s*	15
δ	*m*	0.025
*L*	*m*	0.05
